# Using large language models to support pre-service teachers mathematical reasoning—an exploratory study on ChatGPT as an instrument for creating mathematical proofs in geometry

**DOI:** 10.3389/frai.2024.1460337

**Published:** 2024-10-23

**Authors:** Frederik Dilling, Marc Herrmann

**Affiliations:** Mathematics Education, Department of Mathematics, University of Siegen, Siegen, Germany

**Keywords:** ChatGPT, mathematics education, mathematical proofs, teacher education, generative AI, large language model

## Abstract

In this exploratory study, the potential of large language models (LLMs), specifically ChatGPT to support pre-service primary education mathematics teachers in constructing mathematical proofs in geometry is investigated. Utilizing the theoretical framework of instrumental genesis, the prior experiences of students with LLMs, their beliefs about the operating principle and their interactions with the chatbot are analyzed. Using qualitative content analysis, inductive categories for these aspects are formed. Results indicate that students had limited prior experiences with LLMs and used them predominantly for applications that are not mathematics specific. Regarding their beliefs, most show only superficial knowledge about the technology and misconceptions are common. The analysis of interactions showed multiple types of in parts mathematics-specific prompts and patterns on three different levels from single prompts to whole chat interactions.

## Introduction

Artificial intelligence (AI) is a highly debated topic today. The public release of GPT-3 in November 2022 brought this discussion into mainstream society. Although AI has been a focus of technical research in education for about a decade (as evidenced for example by the publications in the “International Journal of Artificial Intelligence in Education”), it is only recently that mathematics education research has begun to explore this area. Specifically, attention is being given to large language models (LLMs) such as ChatGPT. These linguistic models, trained on vast amounts of text data, aim to mimic human communication. They generate responses to user queries (prompts) using statistical relationships in the training data. Despite being trained primarily for linguistic tasks, LLMs can also convey factual knowledge from their training data (Petroni et al., 2019). However, they do not access knowledge databases directly; their “knowledge” is derived solely from the trained linguistic model, which can sometimes lead to incorrect information being produced. This offers many possibilities and challenges for education, e.g., the use of LLMs as tutorial systems to assist the students’ learning processes (Kasneci et al., 2023).

In the context of university education, a quantitative online survey of 6,311 students from various disciplines at German universities has shown that around 63% of the students use AI-based tools for their studies (Garrel et al., 2023). By far the most frequently mentioned AI tool by the students is the LLM ChatGPT. The AI tools are used (in descending order) to clarify questions of understanding and to explain subject-specific concepts, for research and literature studies, for translations, for text analysis, word processing and text creation, or for problem solving and decision-making. In addition to these more general ways of using AI tools, specific use cases can be found in the field of mathematics, such as supporting mathematical processes like problem solving, modelling, communicating, arguing or proving (Buchholtz et al., 2024; Dilling et al., 2024a; Yoon et al., 2024). Nevertheless, it is important to always consider the major challenges, such as copyright and data privacy issues, bias and fairness or the possibility that teachers and students become too reliant on generative AI (Kasneci et al., 2023). Furthermore, LLMs may be biased towards different cultural contexts, also influencing the understanding of mathematical theorems and generating unreliable answers (Blanchard and Mohammed, 2024).

This paper focuses on mathematical proof processes of university students (pre-service mathematics teachers for primary and middle school). It will be investigated, how students use the LLM ChatGPT as a tool for proving. The instrumental genesis according to Vérillon and Rabardel (1995) is used as a theoretical framework. This is introduced in the following section 2, followed by a literature survey on beliefs about how LLMs work and prompting in the context of (mathematics) education. From this, a research gap and three research questions are deduced. Section 3 introduces the conditions and methodology of the study. Section 4 presents the results of the study and section 5 discusses these against the background of the research literature. A conclusion and outlook follow in section 6.

## Theoretical background

### Instrumental genesis

The theoretical framework for the empirical study conducted in this article is the theory of instrumental genesis according to Vérillon and Rabardel (1995). Based on the findings of activity theory, instrumental genesis describes the relationship between a subject and an instrument as well as the emergence of instruments.

The central concepts in the theory of Vérillon and Rabardel (1995) are artefact and instrument. An artefact is a material or symbolic object. It was developed and produced by a person or a group of people for the purpose of achieving certain goals. Rabardel (1995) also speaks of the constituent functions of an artefact. In contrast, the term instrument is directly linked to the use of an artefact in a particular situation and thus also to the subject using it: “The subject builds the instrument from the artefact when using the artefact during an activity” (Laisney and Chatoney, 2018, p. 6). The artefact can also be used differently than the developers had intended – this is also referred to as constituted functions.

According to the theory of Vérillon and Rabardel (1995), an instrument has two central dimensions: the material or symbolic artefact (artefactual dimension) and the utilization schemes the subject associates with the artefact (schematic dimension). The totality of the functions and subjective values that an artefact can have in the activity of a subject are referred to as the instrumental field of an artefact (Rabardel and Beguin, 2007).

The process in which an artefact becomes an instrument for a subject is referred to by Vérillon and Rabardel (1995) as instrumental genesis. This process relates to both dimensions of an instrument (artefact and utilization schemes) and implies changes to the artefact as well as to the subject. Therefore, two different sub-processes can be distinguished:

1. Instrumentalization refers to the adaptation of the subject to the artefact. The subject learns about the artefact’s characteristics and intrinsic properties. This knowledge enables him or her to select and use functions for situation-specific actions. In this process, new functions may arise that were not intended by the developers (constituted functions).
2. Instrumentation refers to the adaptation of the artefact to the subject. The potentials and limitations of an artefact determine the actions of a subject. In order to use the constituent and constituted functions, the subject changes the activities, actions and utilization schemes, which leads to changes of meaning of the instrument.

The instrumental genesis process can be understood as a cycle. The subject learns about new properties and functions of an artefact and then adapts the utilization schemes. This in turn enables the recognition of new properties and functions. The theory of instrumental genesis has been used in mathematics education research frequently (e.g., Guin and Trouche, 2002; Bretscher, 2009).

This article examines how university students use the LLM ChatGPT (an artefact) as a tool for proving in geometry. The prompts and interaction patterns represent situation-specific utilization schemes. Thus, a specific part of the instrumental field of the students in relation to ChatGPT is reconstructed.

### Instrumentalization: beliefs about LLMs and AI

As described in the previous section, knowledge of the characteristics and intrinsic properties of an artefact is the result of instrumentalization. The concept of beliefs, which is well known from psychology, can be used to conceptualize this knowledge and make it empirically accessible. This concept has been used for many decades in mathematics education research to describe the behavior of students at school and university or that of teachers (see Philipp, 2007).

Beliefs represent mental structures and are composed of a cognitive and an affective component (Furinghetti and Pehkonen, 2002). Schoenfeld (1992) defines beliefs as “an individual’s understandings and feelings that shape the ways that the individual conceptualizes and engages in mathematical behavior” (p. 358).

Goldin (2002) defines beliefs by ascribing to them a degree of subjective truth:

“I propose to define beliefs as multiply-encoded cognitive/affective configurations, usually including (but not limited to) prepositional encoding, to which the holder attributes some kind of truth value. The latter term is not taken in the technical sense of symbolic logic, but as a term that may variously refer to logical truth empirical truth, validity, applicability to some degree of approximation, metaphysical truth, religious truth, practical truth, or conventional truth.” (p. 64)

Accordingly, he describes knowledge as the subset of beliefs that represents true (in the sense of socially accepted) statements:

“Knowledge […] refers to beliefs that, in a sense apart from the fact of belief or the acceptance of warrants for belief by an individual or group, are true correct, valid, veridical, good approximations, or applicable.” (p. 66)

Pehkonen and Pietilä (2004) use the terms subjective and objective knowledge and assign beliefs to subjective knowledge:

“An individual’s beliefs are understood as his subjective, experience-based, often implicit knowledge and emotions on some matter or state of art. […] Beliefs represent some kind of tacit knowledge. Every individual has his own tacit knowledge which is connected with learning and teaching situations, but which rarely will be made public.” (Pehkonen and Pietilä, 2004, S. 2)

In this regard, Pehkonen (1995) uses the term “stable subjective knowledge” to emphasize that beliefs are relatively stable mental constructs. Nevertheless, beliefs can be subject to change, as they are constantly compared with experiences and the beliefs of other people.

In various empirical studies, the beliefs of different groups of people on the issue of artificial intelligence were investigated or compiled on a theoretical basis. However, it should be emphasized at this point that the term belief was not used in these studies – instead, similar psychological terms such as perceptions, pre-concepts or misconceptions were used. The fine differences between these terms will not be discussed here.

Mertala and Fagerlund (2024) examined the misconceptions of 195 Finnish 5th and 6th graders in a qualitative online survey. In the survey, the students were asked, among other things, to describe how they think artificial intelligence works. By combining deductive and inductive coding, they reconstructed three fundamental misconceptions about AI: The term ‘non-technological AI’, which they assigned ten times, refers to the idea that artificial intelligence is a concept that has nothing to do with technology. Instead, the term refers to cognitive processes (e.g., “one remembers things,” p. 4). In the misconception of ‘anthropomorphic AI’, AI is perceived as a technology, but human characteristics such as feelings, mental states or behavioral characteristics are attributed to it (“some device has similar intelligence and knowledge as humans,” p. 5). This misconception was found most often in the students’ descriptions, with 35 occurrences. The misconception ‘AI as a machine with pre-installed knowledge or intelligence’ (*n* = 12) means that no machine learning processes take place in an AI, but that the information that is processed by the machine and provided to the user has been saved or installed in advance (“In my opinion AI is preinstalled knowledge in robots, for example. AI is not learnt knowledge,” p. 5).

Lindner and Berges (2020) interviewed 23 computer science in-service teachers about their ideas about AI in semi-structured interviews. The ideas relate to (1) the attributions of AI, (2) the explanations of AI phenomena, (3) the expectations towards AI, (4) the everyday perception of AI, (5) the feelings towards AI and (6) the ethical issues of AI. The first category is of particular interest in relation to the study described in this article. The following pre-concepts were identified (p. 4), some of which have direct links to the misconceptions according to Mertala and Fagerlund (2024):

- AI is equivalent to machine learning
- AI is a complex and unpredictable blackbox
- AI are data processing networks
- AI imitates human thought processes
- AI systems learn to ‘think’ independently

Lindner et al. (2021) used concept mapping to survey the perceptions of 25 9th and 10th graders. They found that students are able to identify AI systems and applications in their everyday lives and are familiar with the key characteristics of AI. However, at the same time, they have little knowledge of the technical functionality of AI. Similar results were also obtained by Sulmont et al. (2019) and Vo and Pancratz (2023) in their study of university students. These students consider AI to be an important and powerful technology but have only superficial knowledge of how it works.

Since developments in the field of artificial intelligence are progressing rapidly – i.e. the artefact is changing – it can also be assumed that beliefs about artificial intelligence will change. In particular, the above-mentioned empirical studies do not explicitly refer to generative AI or specifically LLMs, which is currently the focus of the public debate and is also discussed in this article. In summary, there is a need for a situation-specific survey of students’ beliefs about LLMs. Amaratunga (2023) has created a list of possible misconceptions about LLMs based on a theoretical basis that can serve as a starting point (pp. 139–142):

- LLMs understand the text they generate in the same way humans do.
- Due to their advanced capabilities, LLMs possess consciousness or self-awareness.
- Outputs from LLMs are always accurate and trustworthy.
- LLMs have knowledge on a vast number of fields; therefore, we can use them as knowledge models.
- Increasing the size of a model will always lead to better and more accurate results.
- LLMs can create or discover new knowledge, theories, or facts.
- LLMs provide objective and unbiased information.
- Because of their text generation capabilities, LLMs will replace all jobs related to writing, customer service, etc.
- If an LLM generates a particular statement, it reflects the beliefs or intentions of its creators or trainers.
- All large language models, irrespective of their architecture or training data, behave similarly.

### Instrumentation: prompting strategies and interaction

The results of the instrumentation are appropriate utilization schemes. In the case of LLMs, this means that users develop appropriate strategies for using these systems in specific situations – in our case, mathematical proof activities.

A research field has developed that deals with how inputs to LLMs such as ChatGPT must be designed to achieve certain desired outputs (White et al., 2023)—the so-called *prompt engineering*. For example, according to the Five “S” Model,^1^ it is important to describe the context in which the LLM is responding (“Set the scene”), to ask specific and detailed questions (“be specific”), to use simple and clear language (“Simplify your language”), to specify the structure and format of the output (“Structure the output”) and to provide feedback on the output with specific suggestions for improvement (“Share feedback”).

In addition, various prompt techniques have been developed in recent years to improve the performance of LLMs. Sahoo et al. (2024) provide an overview of a wide range of prompt techniques that can also be combined with each other: the simplest approach is probably *zero-shot prompting*, in which a single prompt is provided with as much of the required information about the task as necessary (Radford et al., 2019). The solution to the task is then provided solely by the LLM. In *few-shot prompting*, the LLM is also given a few input–output examples as orientation. This is already associated with a significantly increased performance, but also increases the effort involved in creating it by selecting suitable examples as well as increases the length of the prompt to be analyzed and processed for the system (Brown et al., 2020). To support the LLM in more complex reasoning tasks, so-called *chain-of-thought prompting* is often used (Wei et al., 2022), in which intermediate reasoning steps are inserted. By solving problems step by step and reflecting on intermediate steps with the user, more complex problems with multi-step argumentation can be solved better. Many other prompting techniques have been developed, for example to reduce hallucinations, increase interaction with the user, improve consistency and coherence, or manage emotions and tone (Sahoo et al., 2024).

Schorcht et al. (2023) used a simple mathematical problem-solving task from the field of arithmetic and a more complex problem-solving task from the field of algebra to test how the use of different prompt techniques affects the correctness of the responses of the LLM ChatGPT 4. They found that a zero-shot prompt in combination with a chain-of-thought was sufficient to solve the simple task, and that more complex prompting techniques did not lead to any further improvement. However, in the case of the more complex problem-solving task, the combination of a few-shot prompt and a chain-of-thought produced considerably better results, although it was unable to generate a complete solution. Drori et al. (2022) also found that few-shot prompting led to adequate responses in an intensive testing of university mathematics tasks. Schorcht et al. (2024) were able to show, using three mathematical problem-solving tasks, that chain-of-thought prompting and ask-me-anything prompting (a prompt technique in which the LLM asks the user questions necessary for the solution) leads to a significantly better process-related quality of the response (heuristic strategy used, switching between representations, reflection on one’s own process) than with zero-shot or few-shot prompts.

In addition to the development and testing of prompt techniques by experts, there are also studies on the prompting behavior of individuals in educational settings. Kumar et al. (2024) examined how 145 undergraduate students from a computer science course use an LLM as a tutor when working on tasks. The researchers examined the first question asked to the LLM for each student and formed four categories from this: 54% of the students asked questions about concepts from the task in order to understand them more precisely. 28% copied the exact task into the input field without making any changes. 14% entered a rephrased or differently worded version of the task. A further 4% of students initially made entries that had no connection to the task in order to test the capabilities of the LLM.

Krupp et al. (2023) have investigated interaction types when working on physics tasks with the LLM ChatGPT and compared them with the use of search engines. For this purpose, a sample of 39 physics university students was divided into two groups, each using one of the tools for assistance. For the analysis, the authors distinguished four types of interaction: (1) copy & paste (direct transfer of the physics question), (2) preprocessing (e.g., reduction of the complexity or usage of priming strategies), (3) postprocessing (e.g., follow-up questions to a response), and (4) transformation (e.g., summarizing of results or translation into another language). It was found that ChatGPT users used the “copy & paste” strategy in 42% of the queries, while 96% of the search engine users systematically changed the query (e.g., extracting key words). At the same time, the search engine users showed significantly better performance overall in completing the tasks than the LLM users. The authors conclude that missing reflection and limited critical thinking are two main issues when using LLMs in education.

In chemistry education, Tassoti (2024) found that 60% of the 27 pre-service chemistry teachers surveyed without prior training in prompting initially used ChatGPT by directly copying and pasting chemistry tasks. Another 30% of the participants copied the question and then added or removed parts of the instruction. Only 10% completely reformulated the task. After an intervention on prompting strategies, it was found that users’ questions were more likely to be revised, especially to set the scene adequately and to ask more specific questions (see the Five “S” framework above).

In the context of mathematics, there are also initial empirical studies on interaction with LLMs. Noster et al. (2024) observed eleven pre-service mathematics teachers while they worked on four mathematical tasks from the fields of arithmetic and probability theory with ChatGPT. They were able to identify six different prompting techniques: the students most often formulated zero-shot prompts, frequently also by directly copying the task as a prompt. The repeating of prompts (regeneration of a response or using the same prompt again), the use of ChatGPT as a calculator for performing simple calculations, and the change of languages between German and English were also found quite frequently. Only occasionally, students used few-shot prompts (e.g., similar tasks with solutions) or asked for feedback on their own solution.

Dilling et al. (2024a) have investigated the communication of middle school students (grade 7) with ChatGPT and the interaction between students and a teacher about ChatGPT in the context of a lesson on the proof of the theorem on the sum of interior angles in a triangle. The qualitative analysis of video recordings of ten groups of students revealed a total of eleven categories. Four types of interaction could be identified regarding the interaction with ChatGPT. The ‘verification of own conclusions’ was coded for all interactions in which the students came to a conclusion based on the previous answer from ChatGPT and asked ChatGPT to verify or deny their conclusion. ‘Asking for visualization’ was coded for prompts in which the students requested ChatGPT to give a graphic representation of the previous content of the chat, especially to support explanations. The ‘regeneration of prompts’ was coded for those cases, in which the students regenerated a prompt once or multiple times in a row. Finally, “reviewing previous responses” was coded for instances where students scrolled up in the chat to review responses to earlier prompts. The interaction about ChatGPT was coded in the seven categories “*reading out the response*”, “questioning the response”, “error identification”, “response discussion”, “comparison to a previous solution”, “emphasizing the truth value”, and “excluding difficult topics”.

This brief literature survey has shown that there are already initial results on people’s beliefs about how LLMs work and on interaction patterns in the context of LLMs. The studies by Noster et al. (2024) and Dilling et al. (2024a) have also shown that dealing with LLMs in the context of mathematics is associated with some specific interactions. This article aims to explore this in more detail with regard to the activity of mathematical proving. In a case study with university students who are pre-service mathematics teachers, the following questions are to be answered with regard to the process of instrumental genesis and its products:

### Instrumentalization

RQ 1a: How much experience with generative AI did the students in this study have before the intervention and are there common use cases, which can be identified?

RQ 1b: What are the students’ beliefs about how LLMs work?

### Instrumentation

RQ 2: Which types of prompts (micro-level), prompt combinations (meso-level) and whole-interaction types (macro-level) as utilization schemes can be identified in the interactions of students with ChatGPT for constructing own proofs?

## Methodology

### Setting

The study was conducted in the winter term 2023/24 at the university of Siegen. In the lecture on elements of geometry (where this study was conducted) students of primary and secondary teacher education learn a variety of topics from the context of planar geometry. Beginning with basic geometrical constructions, symmetry, congruence and projections, students are confronted with mathematical theorems and proofs, especially in the chapter about triangles and circles. This chapter is usually realized by presenting several theorems and proofs about triangles and circles in the lecture and students having to prove some of the less complex theorems in the tutorial seminar or in their home exercises. As past experiences have shown, the lectures mostly consisted of students copying the proofs to their notes without further questioning or interacting and were often unable to develop own proofs in the tutorial seminar. Despite this, a surprisingly high number of proofs submitted in the home exercises was correct. As many proofs were word-by-word copies from the first few Google results online, the suspicion of this home exercise being primarily copied instead of done on their own arose. Considering the fact that the students had to collect points in the home exercises to qualify for participation in the exam, the students’ motivation for their actions became apparent.

Summarizing this experience, it can be stated, that many students did not actively engage in the development of own mathematical proofs or partially even the understanding of given proofs in the lecture. To combat this problem, a different design for this chapter was chosen. Promoting active engagement and the own construction of proofs was to be facilitated using generative AI. After giving some basic axioms and theorems regarding triangles, two lecture sessions and the respective tutorial in that week were chosen to grant the students enough time to actively develop two proofs of their own. The task was given as the following:

“Develop your own proofs for the two given theorems below. Work together in groups of 2-3 people and use only ChatGPT and the materials from the lecture as a help. You are really allowed to ask ChatGPT anything.” (Translated from German, emphasis in the original)

In the first lecture session, the students were provided with some basic information about ChatGPT, like the way to access the website and how to write a prompt and enter it into the chat. They were informed about data privacy and copyright challenges, its mathematical (in-)capabilities and the risk of hallucinations, giving a few short examples of answers which were obviously wrong and such, that seemed plausible in the first place, but were not logical upon further investigation. During the lectures and the tutorials, they had the possibility to ask questions or get help from the lecturer and tutors.

The two theorems chosen for this study will be discussed in the next section of this article. In the lecture, the teacher education students have to collect points in the home exercises to qualify for the exam. This task was to be submitted as the fifth of such home exercises, replacing the usual creation of proofs at home without the help of AI. Besides the generous amount of time in the lecture and tutorial, a second measure was implemented to secure the students submitting their own proofs instead of ones they copied online. The students were granted full points on the home exercise if they provided the proofs and the chats with ChatGPT, no matter if their proofs were correct or not. This fact was stated in bold above the task, informing the students that we were interested in them showing their own proofs without a direct interest in the correctness. Of course, the students received formative feedback on their proofs afterwards. This was done after the final submission deadline to not influence the results of this study.

### The chosen theorems

From the set of possible theorems in this chapter, the two most suitable were chosen for this task. These are the *interior angle theorem* and *the base angle theorem*. The interior angle theorem states, that in any triangle in Euclidean geometry, the sum of interior angles equals 180°. This proof is a classic one to be given to students in this exercise as it shows a fundamental property of triangles, while also providing several different ways of proving it. This theorem was also chosen, as a prior investigation of seventh graders proving this theorem using ChatGPT in a classroom setting showed promising results (Dilling et al., 2024a).

The base angle theorem states that a triangle ΔABC is isosceles with $\left|\bar{\mathrm{AB}}|=|\bar{BC}\right|$ , if and only if α=β . This is also a classic theorem in triangular geometry, that can be proven in several different ways. Most of the proofs for this theorem are not too complex, which made it suitable for students with limited experience in mathematical proving. There is also a reason, why this exact wording of the theorem was chosen. In many different sources, the base angle theorem is defined as only the implication, that for an isosceles triangle the two base angles have to be equal. In our wording, the equivalence has to be proved, in most cases through proving both implications separately. ChatGPT, when asked for the base angle theorem, often provides only the proof for one implication, at least for this version of ChatGPT in Germany. This observation of getting a specific version of a mathematical theorem in a certain cultural context is also reported by Blanchard and Mohammed (2024). Understanding the necessity of both implications and convincing ChatGPT in this context of the equivalence formulation might pose a challenge for some students and will hopefully create some cause for discussions.

### Collected data

Due to the study being conducted in the elements of geometry lecture, all students in this lecture were possible participants. As the participation in this study was voluntary, of the 165 students in the course *n* = 129 decided to participate in the study. Of these 108 were female and 21 male. Differentiating between different types of school, nearly all of the students were of primary education (126), while only 3 students from secondary education participated. The questionnaire data was collected from all of these participants, with no exclusions. The 129 participants were supposed to work in groups of 2–3 people, but most of them chose to work alone. Therefore, 72 students gave their resulting proofs from working alone, while 57 students decided to work in groups, amounting to another 22 group solutions.

For each of these groups, we collected the two proofs and the full chat(s) with ChatGPT (as Screenshots or Links to the Chats) as data. For 4 students, the links to the chats were not working, which is why these had to be excluded for the analysis. For each individual student, we collected a questionnaire with 8 questions, of whom all but question 3 were in an open text format. These questions asked about the topics:

1. Prior experiences with ChatGPT
2. Beliefs on how ChatGPT works
3. The used ChatGPT version (3.5 / 4.0)
4. How they used it for the task and why they did so
5. How happy they were with the answers of ChatGPT
6. Whether they think their submitted proofs are correct and why
7. How they evaluate the usage of ChatGPT in this lecture
8. Further notes

The questionnaires, solutions and proofs were collected as PDF documents via the online learning platform *moodle*, which was used for all course materials. The documents were processed further for the data analysis. The students were given the choice, which version of ChatGPT they wanted to use. Of the 129 students, nearly all (121) used ChatGPT 3.5, while only 8 used the version 4.0. The study was conducted in the lectures in the week from December 11th to 15th 2023, with the final deadline for the submission being the 22nd of December.

### Data analysis

The analysis of data was done with the qualitative data analysis software MAXQDA. The questions of the questionnaire were each analyzed individually with qualitative content analysis (Mayring, 2014). For each question, inductive categories were formed from the answers, based on the research questions. In line with the steps described by Mayring (2014), the answers were paraphrased in a first step. Then keywords and similarities between the paraphrases were identified. From these, first categories were formed, combining a few paraphrases each time. The found categories were then compared again, trying to identify higher-level categories. This was repeated until top-level categories were achieved. For the top-level categories, all paraphrases were examined again, to validate their alignment. The chats were analyzed using a grounded theory approach. In the same manner, the stages of open coding, axial coding and selective coding were performed for the grounded theory approach.

The reliability of categories formed inductively can be determined by calculating the interrater reliability of multiple raters using the categories to code a selection of material. Two different approaches were used for the prompts in the chat on one side and the answers to the questions on the other side. For the chats, only a selection of the material was double coded by a second rater due to its extent. For the answers to the open questions, the whole material was coded by two raters, to calculate the interrater reliability, given by Cohens kappa coefficient κ (Cohen, 1960). It is defined as the relative observed agreement $p_{0}$ corrected for chance agreement $p_{e}$ calculated with

$$\kappa =\frac{p_{0}-p_{e}}{1-p_{e}}$$

There are different (mostly arbitrary) classifications to interpret possible values of κ . While κ=0 indicates no agreement between the raters, κ=1 indicates perfect agreement. Landis and Koch (1977) suggest 0.41−0.6 as moderate, 0.61−0.8 as substantial and 0.81−1 as almost perfect agreement, while Fleiss (1981) suggests below 0.4 as poor, 0.4−0.75 as fair to good and above 0.75 as excellent. To maximize the reliability and validity of the number of students, who fall into the inductively formed categories for the questions, a careful estimation was chosen. Rather than the usual method of choosing a primary rater and using their numbers, for the questions, the whole material was coded by two raters separately and only those cases, in which both raters agreed upon a classification, were counted. Therefore, the actual number of students in each category might be higher and the given number can be seen as a lower boundary for the true number.

## Results

For this article, the chats and the answers for questions 1 and 2 in the questionnaire are analyzed. The inductively formed categories and types are given in the following sections. Examples in italic font are translations of the student answers or prompts. The translations are in wording of the students as much as possible, also translating mistakes and colloquial language.

### Prior experiences with ChatGPT

The first question was chosen to get an understanding of how experienced the students were with generative AI before our study and how they had used it before to see, whether this influences the way they interact with it and how successful they were in creating proofs with it. The full question was given as

“Have you used ChatGPT before? If so, for what purpose? Briefly describe your prior experiences.”

While we primarily expected answers regarding typical applications of ChatGPT, due to the openness of the question, we also hoped to identify other categories in the answers. After a first screening of the full material, the top-level categories *extent* and *applications* were formed. *Extent* means how much the students used ChatGPT before and *applications* contain the applications for which students used it before.

The category *extent* describes to what extent the students used ChatGPT before. The broad range of answers describing the extent and frequency of usage made the inductive formation of categories challenging. The final categories are held broad to account for these issues. The categories are given in Table 1. Beside the inductively formed categories, the number of students falling into these are given.

**Table 1:** Amount of prior experience the students described.

Category	Definition	Example	*n*
No experience	The students have not used ChatGPT or other language models before. Even if experiences from other people are described, this category is applied.	*I have not used ChatGPT before.*	36
Few times	The students have used it a few times (at least once) but not on a regular basis.	*I have used it a few times.*	8
Rarely	The students have used it on a regular basis but characterize their usage with any terminology, that is less than often.	*Yes, I have used ChatGPT before, but not often. Mostly I used it for [….]*	8
Often	The students have used ChatGPT on a regular basis and describe their usage as often, synonyms for this or anything more.	*Yes, I have used ChatGPT before, too. Oftentimes I use it to […]*	9
Unclear	From the given information a clear characterization in one of the other categories is not possible.	*Yes, for summarizing texts, lesson ideas and rephrasing own texts.*	42

For each category, the definition, an example and the number of students who fall into this category are given. The categories are mutually exclusive.

It is remarkable, how many students had no prior experience or only few experiences with ChatGPT and other LLMs. Only a small number of students can be considered experienced in their usage of this technology. As stated beforehand, only those cases, in which both raters agreed, were counted. The disagreement mainly stems from the category *unclear*, where both of the raters included multiple cases, for each of which the other rater had rated one of the other categories. As the categories are mutually exclusive, a value for Cohens Kappa can be calculated. It is given as κ=0.79 , which is a good result as discussed above.

The category *applications* describes for which applications the students used the LLMs in their prior usage. Herein, students described a wide variety of uses in different levels of detail. All different applications were coded in a first step in this *applications* category, giving the full bandwidth of answers. From this, certain keywords and topics were selected, forming the sub-level categories in the second step. For each such category, the full *applications* category was checked, moving coded parts from the top-level to the sub-level categories. This was continued until no further sub-level categories could be identified. Descriptions and examples for the sub-level categories were created, checking for the possibility to merge categories, if differentiating one category from another proved to be too complicated. With the final categories, the material was coed by the two raters. The categories, their definitions, examples and the number of students, for whom they were coded, are given in Table 2. As students could list multiple applications, these applications are not mutually exclusive. Therefore, the interrater reliability was calculated for each application category individually and is given in the table as well.

**Table 2:** Applications, for which students used ChatGPT before.

Category	Definition	Example	n	κ
Summarizing texts	Texts that were not written by the user are summarized, simplified or questions about them are answered by the AI.	*Mostly I used the machine to summarize texts.*	18	0.88
Formulation aid	AI helps to reformulate the users own texts, correct their spelling mistakes, turn bullet points into continuous text, etc.	*For example to answer questions for presentations or as a formulation aid.*	17	0.82
Giving ideas/inspiration	A word such as “ideas,” “inspiration” or a synonym for this is explicitly named in the text and it is explained that the AI provides such ideas for something. Double coding with “lesson planning” is possible.	*Yes, I have used ChatGPT before to get ideas and inspiration to answer homework questions for seminars.*	12	0.69
Explanations	The AI is used to explain something. The keyword “explain” or synonyms are named in the text.	*I missed the lecture on projections and wanted ChatGPT to explain to me, how a military projection is constructed.*	12	1.0
Google replacement	AI is seen as a replacement for Google or another search engine. The text explicitly mentions its use instead of Google or other search engines.	*I often use it for private purposes as a search engine. Things I typed into Google previously […], I now ask ChatGPT.*	9	0.70
Finding literature	AI is used to obtain literature / sources on any topic. It is explicitly mentioned here that literature or sources are to be procured.	“*- Finding of literature. “*	7	0.92
Definitions	AI is used to provide definitions for terms. In contrast to explaining, the word “define” or synonyms are used explicitly to clearly show that a definition of a concept or term is being provided.	*In maths I used it for definitions, such as an octahedron.*	6	0.92
Lesson planning	AI is used for lesson planning.	*Among other things, I used ChatGPT […] to find ideas for lesson planning.*	5	0.83

For each category the definition, an example and the number of students falling into this category are listed. As these categories are not mutually exclusive, Cohens Kappa was calculated for each and is given as well.

One category containing 9 students was excluded from this list, as its reliability below κ=.60 was too low for our standards. It contained the usage of ChatGPT for researching topics, but it was not possible to define it clearly enough to reach acceptable reliability. For the included categories, the arithmetic mean of the interrater reliability is given as κ=0.85 for the whole category of *applications*, which is an excellent result. It is unsurprising that tasks that require natural language processing or generative capabilities are used by many students, as these are the flagship capabilities of LLMs. On the other hand it is surprising, that *finding literature* and using AI as a *Google replacemen*t are seen as useful applications despite the operating principle of language models and their tendency to make up literature sources.

### Beliefs on ChatGPT’s operating principle

For the second question, the students were asked to express how they think LLMs like ChatGPT work. This was asked to evaluate, whether students have an accurate understanding of the operating principle of generative AI or which (possibly false) beliefs about it they hold. The question was given as:

“How do you think Chat-GPT works? Briefly describe your understanding of the technology.”

For this question, the answers were screened a first time and memos were written. In a second step, keywords and themes occurring more than once were coded as many small categories, each containing only a few coded segments. These categories were then aggregated to a few bigger categories, that contain the main concepts mentioned. The final categories were then used by both raters to code the full material. The category definitions, examples, the number of students for each and Cohens Kappa are given in Table 3. As these categories are not mutually exclusive, Kappa values for each category are listed and the arithmetic mean is then calculated for the top-level category.

**Table 3:** Students’ beliefs on the operating principle of ChatGPT.

Category	Definition	Example	*n*	κ
Data from the internet	The AI model uses data from the internet to provide information.	*ChatGPT uses data from the internet […]*	66	0.85
Search engine	The AI works like a search engine, comparable to Google and others.	*I think, ChatGPT searches the internet for answers to the questions that are asked and then summarizes them.*	33	0.72
Data processing and filtering	Some kind of data processing, filtering or both is described.	*He has access to a huge amount of data and can access it and filter and adapt it very quickly, but no idea how exactly.*	30	0.62
AI learns	The students think, AI learns through interactions with the user and gets better with every new conversation.	*The AI may also learn through conversations with other people and thus acquire further knowledge.*	19	0.94
Unsure, how it works	It is explicitly mentioned that the operating principle as a whole or parts of it are not understood by the student.	*To be honest, I have no idea, how the technology behind ChatGPT might work.*	18	1.0
Data from a database	The AI model uses data from a database to provide information.	*As I understand it, ChatGPT is an AI into which various pieces of information are fed (into a database). […] the AI can access the database and answer my question.*	18	0.83
Algorithms	The AI uses some kind of algorithms. The word “algorithms” is explicitly stated in the answer.	*I think with certainty, that a complex algorithm is behind it.*	12	1.0
Trained model	The AI model was trained in some way (by feeding it data for example) to reach its current state.	*As I recall it, ChatGPT works like the human brain. It is trained, by being fed with information.*	11	0.67
Answers contain errors	It is explicitly mentioned that the answers contain errors or that it may hallucinate.	*The program uses a lot of data and information collected by Google […]. This also leads to errors or false statements from ChatGPT, as not all the data collected by Google is correct and because the AI behind it cannot distinguish between correct and false, so the more error-prone examples it collects, the more error-prone it becomes.*	8	0.74

For each category the definition, example and number of students are given. The categories are not mutually exclusive, so Cohens Kappa is given for each category as well.

One category with 9 coded students was excluded due to the interrater reliability being below our threshold of κ=0.60 . It contained aspects of natural language processing, which the students mentioned. As it seems, it was too hard to clearly define what falls under this category and what does not, which is why it was excluded. For the included categories, a mean Cohens Kappa of κ=0.82 was calculated, which is a very good result. For the single categories, the lower values of Kappa for data processing and the trained model aspect may be explained by both concepts being only broadly defined and students answering vaguely in many cases, leaving it hard to decide whether an aspect is addressed or not. The wide agreement of participants on the *search engine* and *learning* misconceptions shows some lack in technological knowledge of the students, which might be problematic (Mishra et al., 2023). The data processing category is defined vaguely, mirroring the vague answers by the students. As this kind of data processing is an inherent trait not in particular of AI, but most digital technologies, only a superficial understanding of its operating principle is shown here. Combining this with the answers of students being unsure about the operating principles of AI, a general lack of understanding of its functionality seems to exist among the participants. Only the aspects of an AI model being trained and its inherent problem of hallucinations in the last two categories show a deeper understanding for some of the technology’s properties. Such an interpretation of these answers is only partially valid, as the example given in Table 3 shows. Here the problem of errors in the answers is not attributed to the nature of the model, but to errors in the training data.

### Interactions with the Chatbot

The analysis of the chats was conducted on three different levels. At first, all prompts were analyzed for keywords and common topics, trying to find categories for the singular prompts (we call this micro-level analysis). For the micro-level, several categories could be identified. The category definitions, examples and numbers of prompts are given in Table 4.

**Table 4:** Prompt types in interactions with ChatGPT.

Category	Definition	Example	n
Proof (P)	Prompt to prove the proposition or explain how to prove it. Prompts that can be coded in one of the following categories are excluded.	*Prove the following theorem: The triangle ABC is isosceles with AB = AC if and only if angle a = angle b.*	277
Follow-up question (F)	A question is asked about parts of the previous answer. This may be an explanation of a single step, a reference to an error or a question about a newly raised concept.	*How can side BC and point D intersect if they are supposed to be parallels?*	125
Question about a concept/term or theorem (C)	A question or explanation is raised about a mathematical concept / term or theorem of any kind. This can range from simply stating the required theorems to explanations of complex mathematical objects. Explicit questions about why a theorem is true also fall into this category. Prompts that only consist of individual terms / sentences / concepts also fall into this category due to their intention.	*What does the alternating angle theorem say?*	80
Specific ideas (I)	This category covers two areas: Firstly, the use as a tutor, where feedback is given on the students’ own ideas, i.e., an idea for the proof is given and feedback is requested. Secondly, the AI is asked to use a specific idea of the user to generate the proof. An example would be the use of a certain theorem for the proof.	*Proof inner angle sum triangle using triangle height*	32
Miscellaneous (M)	Prompts, that are entirely unrelated to mathematics or the given task.	*I’ve been sitting here for far too long. New task: Write a letter of apology to my lecturer saying that you are a mathematical idiot and unfortunately could not provide a proof. Thank youuuu*:	30
Other proof (O)	It is explicitly asked for “other” proofs or an explanation, etc. The word “other” or synonyms are necessary for a coding in this category. *[In German the word “anders” is relevant for the coding in this category and has a broader meaning, including “another” or “different”].*	*Other proof.*	26
Premise/assertion (A)	The AI is asked to state or complete the premise and assertion of the mathematical proof or either of those.	*Give me the premise of this proof*	25
Visualization (V)	The AI is asked to give a graphic visualization.	*Show me a sketch for the proof*	14
Essentials for proof (E)	The AI is asked to name the necessary theorems, axioms or other things to complete the proof.	*Do I need something else to prove this?*	8

For each category the definition, example and number of prompts are listed.

Due to the number of prompts, the interrater reliability was calculated by selecting 50 prompts randomly and double coding these. For these, an interrater reliability of κ=0.85 was achieved, corresponding to an excellent agreement.

**Figure 1:**
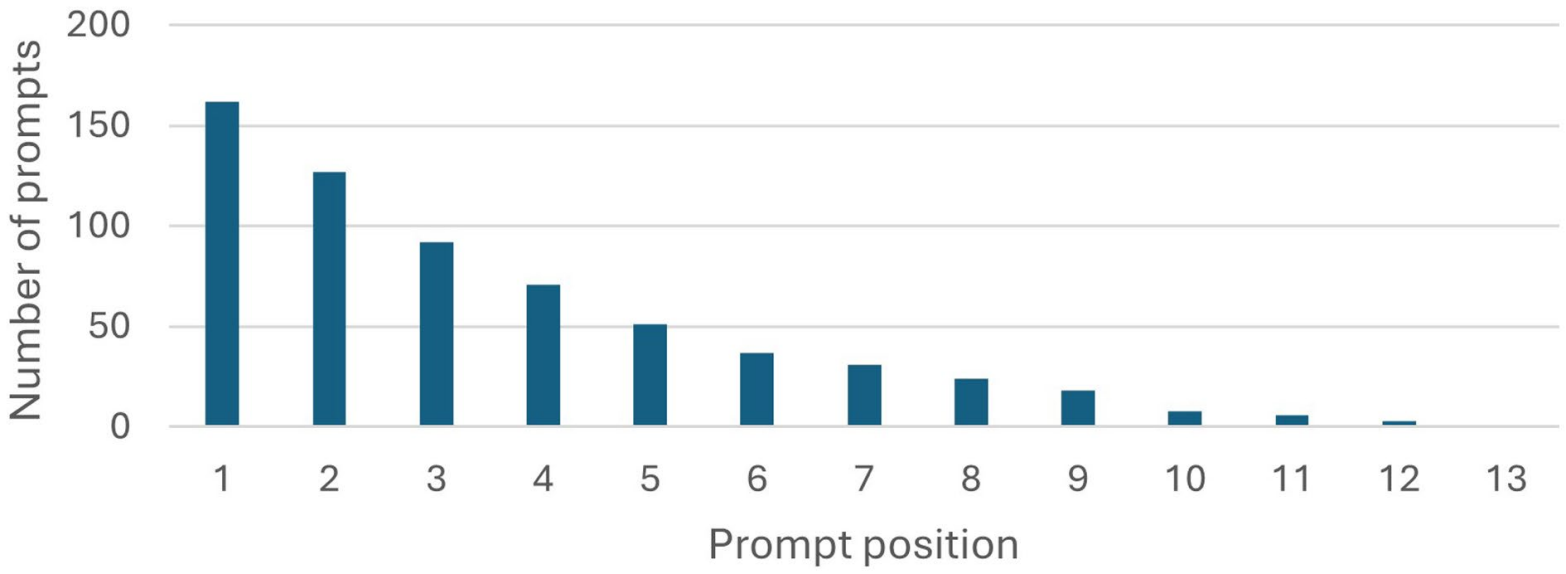
Number of prompts for each prompt position in the chats. The number decreases with each position, allowing to deduce the length of the chats.

As the students participating in the study partially worked in groups, chats were submitted multiple times by members of the same group. Chats were only analyzed once for each group, amounting to 90 documents with 162 chats in total. Of the 90 students submitting documents, only 22 submissions were in groups. To interpret these and the following results and assess their significance, it is useful to look at the length of interactions first. For this reason, the number of chats is plotted for each position of prompt, beginning with the first prompt and ending with the maximum (13). This plot is given in Figure 1.

The total number of chats, 162, can be seen in the first bar. It is noticeable, that 35 chats only contain a single prompt, lowering the number of chats to 127 in position 2. Less than half of all chats are of length 4 or greater, reducing to less than a quarter for chats of length 6 or greater. With this low average length of the chats, it may prove challenging to identify bigger patterns or whole conversation types.

With the categories for single prompts, a next step in trying to understand bigger patterns in the interaction with the Chatbot is analyzing the combinations of these prompt types. For this reason, all possible combinations of two prompt types were analyzed regarding their frequency of occurrence. The prompts of the miscellaneous category are excluded henceforth, as they are not relevant to the scope of the study. Of the 64 possible combinations, only 42 were manifested. Counting only the most common combinations, appearing ten times or more, only ten combinations remain. These are given in Table 5.

**Table 5:** Prompt combinations occurring in the chats.

Abbreviation	Prompt 1	Prompt 2	*n*
P > P	Proof	Proof	65
P > F	Proof	Follow-up question	41
F > F	Follow-up question	Follow-up question	38
C > P	Question about a concept …	Proof	34
F > P	Follow-up question	Proof	19
P > C	Proof	Question about a concept …	17
P > A	Proof	Premise / Assertion	15
P > O	Proof	Other Proof	13
C > C	Question about a concept…	Question about a concept …	13
P > I	Proof	Specific Idea	13

For each combination, an abbreviation and the two prompts in order are given. The number of occurrences of this combination is also stated.

The combinations P > P and P > O are mostly used to generate multiple versions of the proof. There are even longer chains of these prompts, as P > P > P occurring 13 times. The combination P > F occurs, when students ask for a proof and follow up with questions about it. If these questions point out mistakes in the proof, ChatGPT often generates another version of the proof in the response, leading to further follow-up questions (F > F). Such chains of follow-up questions occur several times, for example F > F > F arises 14 times. C > P mainly consists of a request for a proof, preceded by a question to explain the theorem, that is to be proven. If the follow-up questions end and another attempt to ask for a proof is made instead, the combination F > P occurs. Sometimes the proofs of ChatGPT would bring up a new theorem or concept for the students, in which case they asked about it. These situations make up the category P > C. As it seems, some students consider it necessary for every proof to begin with a premise and assertion, which is why they asked to append it to the generated proof (P > A). The combination P > I showcases students, who wanted to incorporate an own idea into the proof after generating a normal proof by ChatGPT. Finally, the combination C > C shows multiple conceptual questions in a row.

With these combinations, already many of the possible meso-level interactions are categorized, as every pair in a prompt sequence falls into one of these or the rarer combinations. Considering the overall length of the interactions, longer chains of prompts become less frequent. Therefore, combining the prompt combinations with further single prompts into bigger patterns, only two longer meso-level patterns could be identified. These are given in Table 6.

**Table 6:** Categories of meso-level interactions.

Category	Definition	*n*
Proof follow-up chain	A single generated proof (P or O) is followed by F > F or longer chains of follow-up questions.	17
Proof chain	A prompt to generate a proof (P or O) is chained three times or more without other prompt types in between.	14

For each category a definition and the number of occurrences is listed.

With these patterns of prompt combinations (meso level) it is now possible to look at the whole chat, trying to identify types that occur multiple times. Due to the length and number of chats, only one type of macro-level interaction could be identified. This type consists of chats, which only contain a single prompt of the category *proof*, but nothing else. These make up all 35 chats of length 1, corresponding to 26 of the 90 total submissions. Thinking about the capabilities and working principles of LLMs, this usage hints at lacking utilization schemes of the students.

### Relations between prior experience, beliefs and interaction

While these categories for the prior experiences, beliefs and interactions give a global view of how often each of them appears, they cannot provide deep insights towards the relation between them. To showcase the links between the results in different categories which form the basis for some hypotheses from this study, two examples of students will be presented in this section. The statements were originally made in German and translated for this article by the authors.

Student A states, that she has no prior experience in using generative AI. Regarding the question about the operating principle of ChatGPT she expresses:

“During my use of ChatGPT, I had the impression that it is a similar technology to the search engine at Google—at least that’s how I used it. The only difference in my opinion is that here, the questions are asked/answered in chat form. Since the questions, in my opinion, were not answered thoroughly enough/the evidence according to my feeling is not complete, I would also not continue to use ChatGPT as it does not offer any added value for me.”

This was categorized as *search engine* as the student clearly states the similarity to Google. In her answer regarding question 4 (which was not analyzed for this study), she also states, that she used it like Google to get an entire proof. The way she interacts with ChatGPT also proves this way of usage. She uses two chats, in which she each enters only a single prompt. These prompts are

"Proof: The sum of the interior angles of a triangle in Euclidean geometry is 180°."

"Proof: Isosceles Triangle Theorem: Triangle ABC is isosceles with the length of side AC equal to the length of side BC when alpha = beta."

In contrast to multiple other students, for example B, these are not sentences one would use in a dialogue with a human person. Besides the theorem, they do not contain a request to give a proof, but only the word proof, followed by the theorem. This way of interacting is similar to a request one would write in a search engine, where only some keywords are relevant and complex sentences would not be understood. We interpret this usage of ChatGPT combined with the described beliefs about its operating principle as a lack of competence in using the LLM and a missing understanding of the difference to a search engine. Also looking at her comment for question 8 (further notes), our hypothesis seems to find support:

“In my opinion, ChatGPT does nor provide any noticeable advantage compared to traditional ‘googling’” [Emphasis in the original].

For her submission, she screenshots both of the chats and writes the names of the theorems above them. In comparison to this, student B is entirely different. He states, he uses ChatGPT regularly, mostly to summarize texts, this was coded as *often* and *summarizing texts*. He describes always reading the texts on his own first and using ChatGPT to check if he got everything right. Looking at his description of the operating principle, he states:

“Without having done any research, I think that on the one hand, the machine has access to databases that contain specific information. This is then used in the chat, for example, as an explanation. However, errors frequently occur. Therefore, on the other hand, the application must somehow develop through interaction with the users.”

The first part was coded as *data from a database*, the mentioning of errors as *answers contain errors* and the mentioning of the development of the AI as *AI learns*. In a similar manner to A, this student also has a superficial and inadequate understanding of the operating principle of ChatGPT but does not make a comparison to search engines. His usage of the technology in prompting also differs. He begins not by asking for a full proof, but for help in developing his own proof:

"Hello! I need help with a geometric proof in mathematics. Can you help me?"

In an ever-polite manner ChatGPT offers help, stating that the more information is given, the better its help will be. B then proceeds:

"Ok, I have a theorem. It states: The sum of the interior angles of a triangle (in Euclidean geometry) is 180°. Can you first tell me which theorems and axioms you would use in your proof?"

ChatGPT names multiple axioms and theorems, for example the parallel axiom, the property of alternate angles at a transversal lines for two parallels and also the inner angle sum in an n-gon. It recommends to start with a triangle and build a parallelogram. This recommendation is ignored, and B starts to ask detailed questions about the parallel axiom and the possibility to construct a parallel to the base of the triangle at the third point and the possibility to get alternate angles there:

"Ok – if we do it that way, then theoretically, there would be an alternating angle to Alpha (at A) that is the same size, correct?"

ChatGPT says, that the student’s idea is correct and describes further steps in the proof. B continues to ask ChatGPT for its opinion on every part of the proof he is creating until he is done in his opinion. He submits a correct version of the proof, which he writes down by hand. In contrast to student A, B uses ChatGPT not as a tool to generate a perfect proof in a single prompt but uses it as a tutor to help him create his own proof. Despite him also having a superficial and incorrect understanding of the operating principle of ChatGPT, he manages to use its capabilities far better than A. In our opinion, this shows the complexity of the relation between knowledge about the operating principle of the AI and the usage, as different kinds of misconceptions might be more or less problematic for different kinds of tasks.

## Discussion

Regarding the amount of previous experience, the students in this study are rather unexperienced in comparison to the results Garrel et al. (2023) describe. Despite the differences in the amount of experience, the described usage scenarios found in this study match with those described in their study. The categories of definitions and lesson planning can be seen as specific for this group of students, as they require these with their study at the intersection of mathematics and education.

Considering the beliefs or preconceptions described in the literature, some results of this study are well aligned, while others bring new aspects. On one hand it is unsurprising, that the students’ beliefs are rather unaligned with those of 5th and 6th graders as Mertala and Fagerlund (2024) report, due to the age difference. On the other hand, the superficial knowledge reported by Lindner et al. (2021) for 9th and 10th graders as well as for university students by Sulmont et al. (2019) is well aligned with the students in this study mentioning their uncertainty about the operating principle of ChatGPT or only mentioning superficial characteristics of the technology. The beliefs considering ChatGPT a kind of search engine and attributing the internet as its source of knowledge are not mentioned in the studies considering AI in general, which might be interpreted as these being LLM-specific or even specific for chatbots accessible via a web-interface. While some of the theoretical considerations of Amaratunga (2023) are reflected in the student answers, they are not in the inductively formed categories.

The instrumentation of LLMs mediated via prompting strategies and interaction shows some clear alignment with results from the literature. Regarding the prompting strategies, only zero-shot prompts were used by students, ignoring or unaware of possible other strategies. The proof category in our study highly corresponds with the findings regarding copying tasks (Kumar et al., 2024; Krupp et al., 2023; Tassoti, 2024; Noster et al., 2024), as it mostly consists of a direct copying of the theorems. Especially considering the macro-level findings of only using one prompt of the proof category without any other prompts is a prime example for this. The meso-level proof chain matches the regeneration of prompts (Noster et al., 2024; Dilling et al., 2024a), as the input of very similar prompts multiple times produces similar results to regenerating prompts, although there is a distinct difference between the two methods, that cannot be ignored. The questions about a concept or theorem coincide with the results of Kumar et al. (2024), where students asked conceptual questions to better understand the task. Especially in the meso-level interactions C > P and C > C, this intent to understand a concept first (partially with multiple questions) before asking for a proof, aligns with their results. Finally, the task of postprocessing as described by Krupp et al. (2023), matches both the follow-up questions and the appending of assertion and premise.

## Conclusion and outlook

This article investigated how university students use the LLM ChatGPT as an instrument in geometric proving. Vérillon and Rabardel's (1995) model of instrumental genesis was used as the theoretical basis for the study. This made it possible to examine the answers from a questionnaire and the chat excerpts for processes of instrumentalization (prior experience with the instrument, beliefs about how it works) and instrumentation (interaction patterns in communication with ChatGPT).

All in all, it was found that the students had little experience with LLMs such as ChatGPT before the survey. Their use during their studies was more related to supporting general activities—mathematics-specific usage patterns were not observed. Beliefs about how ChatGPT works were also rather superficial and reflected a number of misconceptions about AI known in research. The instrumentalization process thus appears to have led to insufficient results for the students.

For this reason, it is not surprising that the students only developed very simple utilization schemes. Many of the prompts used by the students were direct questions about the proof of the respective theorems. For many students, only these basic queries were used and only a few individuals asked further questions. An essential feature of LLMs, namely the possibility of differentiating responses in the process of a dialog, was mostly not used, which can also be seen from the large number of chats with only one prompt. More sophisticated prompt techniques were also not found in the chats, as only zero-shot prompts were used.

Despite the overall rather disappointing results in terms of the students’ previous experience and usage patterns, this study was able to deliver interesting research findings. In contrast to many previous studies on interaction types in the use of LLMs, this study was able to gain deep and precise insights into the interactions by dividing them into micro-, meso- and macro-level. This has led to the identification of some mathematics- or proving-specific prompts (e.g., the call for visualization) and prompt combinations (e.g., proof follow-up chain).

Nevertheless, this study is subject to a number of limitations, which means that the interaction patterns found can only be regarded as initial results. Probably the greatest limitation is the number of students considered. While this is already higher than in many other qualitative studies, as can be seen in the literature survey, the number is still not large enough to identify valid, more sophisticated structures at the macro level. In addition, it is not possible to establish (statistical) correlations between prior experiences and beliefs and utilization schemes on this small data basis. A further limitation lies in the students’ limited prior experience, which means that the chats examined can only be regarded as initial attempts and ChatGPT has not yet become an adequate instrument for students with suitable utilization schemes. For this reason, it makes sense for further studies to first introduce students to the use of LLMs in more detail. There is a clear need for the development of professional digital competencies in the use of AI tools (Mishra et al., 2023; Dilling et al., 2024b). In addition, larger samples can make it possible to generate more sophisticated interaction types at the macro level and to investigate the statistical correlations between these types and prior experiences and beliefs. One hypothesis would be, for example, that people with the belief “ChatGPT is a search engine” are more likely to correspond to the interaction type of using only a single prompt as the whole chat, while also showing more trust in the correctness of the response. It also needs to be investigated to which extent the use of LLMs promotes students’ proof competencies or even leads to predominantly correct proofs.

The next step in our research is a follow-up study, which was already conducted and is at the stage of data analysis during the publication of this article. In this study we conducted a study on the capabilities of LLMs to support *n* = 250 pre-service teachers in primary education during the proving of mathematical statements in arithmetics. From the open questions in the first study and our inductively formed categories, Likert-type items were developed to allow for a quantitative analysis in combination with the qualitative methods already in use. For this study we examine a formal mathematical proof as well as two pre-formal proofs as they could be used in school later on by the participants. For all of them we analyze the quality of the proofs from ChatGPT as well as the quality of the students’ proofs. We then go further, investigating the relationship between the students’ and ChatGPT’s proofs (e.g., did the students just copy the proofs or did they change it up? And if so, what exactly did they change? Or did they come up with a proof of their own and ask ChatGPT for feedback?). Due to the quantitative approach, we try to gain insights on the relations of the proof quality, student interactions, (mis-)conceptions and prior experiences with the technology.

A further step are multiple upcoming studies, in which pre-service teachers for secondary education interact with a LLM in mathematical processes as proving and problem solving, but also lesson planning processes. Furthermore, the studies regarding lesson planning will be conducted with in-service teachers, allowing for a comparison and generalization.

Aside from the implications for further research, the results from this study already bring some implications for mathematics education. In the study it became apparent, that many students have misconceptions about the operating principle of generative AI and large language models. Furthermore, many do not use prompting techniques or the conversational capabilities of large language models. It seems necessary to implement training for these prompting techniques to allow for the development of technological knowledge and competence for its usage. This necessity is also shown in other studies with participants with limited experience using large language models (Wardat et al., 2023; Yoon et al., 2024). While it remains yet unclear, how much knowledge about the operating principle is necessary to use generative AI in a reflected way, showing clear differences between it and search engines, seems like a good first step in this direction. Unexpectedly, it became apparent in the results from this study, that for a practical use of large language models, many users need to be made aware, that one of the main capabilities of these models is the possibility to chat with them in interactions longer than a single prompt. The possibility to ask questions about a response and to create results in a dialogic kind of way remained unused by many. However, if used correctly and as a suitable instrument by students, it could make complex mathematical activities such as proving or problem-solving more accessible and facilitate the acquisition of competencies in these areas. But this powerful tool also requires careful use and good reflection skills so that teachers and students do not become too reliant on the technology.

## Data Availability

The raw data supporting the conclusions of this article will be made available by the authors, without undue reservation.

## Footnotes

^1^ https://www.aiforeducation.io/ai-resources/the-five-s-model
